# Association of the GALNT2 gene polymorphisms and several environmental factors with serum lipid levels in the Mulao and Han populations

**DOI:** 10.1186/1476-511X-10-160

**Published:** 2011-09-20

**Authors:** Qing Li, Rui-Xing Yin, Ting-Ting Yan, Lin Miao, Xiao-Li Cao, Xi-Jiang Hu, Lynn Htet Htet Aung, Dong-Feng Wu, Jin-Zhen Wu, Wei-Xiong Lin

**Affiliations:** 1 Department of Cardiology, Institute of Cardiovascular Diseases, the First Affiliated Hospital, Guangxi Medical University, Nanning, Guangxi, People's Republic of China; 2 Department of Molecular Biology, Medical Scientific Research Center, 22 Shuangyong Road, Nanning 530021, Guangxi, People's Republic of China

## Abstract

**Background:**

The association of UDP-N-acetyl-alpha-D-galactosamine: polypeptide N-acetylgalactosaminyltransferase 2 gene (*GALNT2*) single nucleotide polymorphisms (SNPs) and serum lipid profiles in the general population is not well known. The present study was undertaken to detect the association of *GALNT2 *polymorphisms and several environmental factors with serum lipid levels in the Guangxi Mulao and Han populations.

**Method:**

A total of 775 subjects of Mulao nationality and 699 participants of Han nationality were randomly selected from our stratified randomized cluster samples. Genotyping of the *GALNT2 *rs2144300 and rs4846914 SNPs was performed by polymerase chain reaction and restriction fragment length polymorphism combined with gel electrophoresis, and then confirmed by direct sequencing.

**Results:**

There were no significant differences in the genotypic and allelic frequencies of both SNPs between the two ethnic groups, or between the males and females. The subjects with TT genotype of rs2144300 in Mulao had lower serum triglyceride (TG) levels than the subjects with CC genotype in females (*P *< 0.01). The participants with CT/TT genotype of rs2144300 in Han had lower TG and apolipoprotein (Apo) B levels, and higher high-density lipoprotein cholesterol (HDL-C), ApoA1 levels and the ratio of ApoA1 to ApoB in males; and higher low-density lipoprotein cholesterol (LDL-C) and ApoB levels in females than the participants with CC genotype (*P *< 0.05-0.001). The individuals with GA/AA genotype of rs4846914 in Mulao had higher total cholesterol (TC) and LDL-C levels than the individuals with GG genotype in males (*P *< 0.05 for each). The subjects with AA genotype of rs4846914 in Han had higher LDL-C and ApoB levels, and lower HDL-C levels and the ratio of ApoA1 to ApoB than the subjects with GG genotype (*P *< 0.05 for each). The levels of TC in Mulao were correlated with the genotypes of rs4846914 in males (*P *< 0.05). The levels of ApoA1 in Han were correlated with the genotypes of both SNPs, and the levels of HDL-C and ApoB and the ratio of ApoA1 to ApoB were associated with the genotypes of rs2144300 in males (*P *< 0.05-0.001). The levels of LDL-C in Han were correlated with the genotypes of rs4846914 in females (*P *< 0.05). Serum lipid parameters were also correlated with several enviromental factors.

**Conclusions:**

The associations of both *GALNT2 *rs2144300 and rs4846914 SNPs and serum lipid levels are different in the Mulao and Han populations. These discrepancies might partly result from different *GALNT2 *gene-enviromental interactions.

## Introduction

Prospective epidemiological studies have shown that unfavorable serum lipid levels such as raised levels of total cholesterol (TC) [1], triglyceride (TG) [2], low-density lipoprotein cholesterol (LDL-C) [3], and apolipoprotein (Apo) B [4], together with decreased levels of ApoA1 [4] and high-density lipoprotein cholesterol (HDL-C) [5] are the most important risk factors for coronary artery disease (CAD) and are the targets for therapeutic intervention [6]. It is well recognized that dyslipidemia is a complex trait caused by multiple environmental and genetic factors [6-8] and their interactions [9,10]. Family history and twin studies have shown that genetic polymorphism could account for 40-60% of the interindividual variation in plasma lipid phenotypes [11-13].

Recent genome-wide association (GWA) studies have identified new genetic determinants of several complex quantitative traits, including dyslipidemia [14-17]. These studies evaluated large samples of normolipidemic individuals and showed that several new single nucleotide polymorphisms (SNPs) had replicable modest associations with plasma concentrations of TC, TG, LDL-C, and HDL-C [14-17]. One of these newly identified SNPs is the UDP-N-acetyl-alpha-D-galactosamine: polypeptide N-acetylgalactosaminyltransferase 2 gene (*GALNT2*) [15,17]. *GALNT2 *is a member of a family of GalNAc-transferases, which transfer an N-acetyl galactosamine to the hydroxyl group of a serine/threonine residue in the first step of O-linked oligosaccharide biosynthesis [18]. It has been known that lecithin-cholesterol acyltransferase (LCAT), ApoC3, very low-density lipoprotein, and low-density lipoprotein receptors are all O-glycosylated [19]. *GALNT2 *is a gene in the mapped locus on chromosome 1q42 within 150 kb of the lead SNP, which is located in an intron of the gene [6]. The *GALNT2 *polymorphisms have been found to be associated with alterations of plasma or serum HDL-C [17,19-25] and TG [15,17,24-28] concentrations in some GWA studies but not in others [29-31]. Thus, further studies will be required to characterize the full impact of these SNPs on lipid metabolism.

There are 56 ethnic groups in China. Han nationality is the largest ethnic group, and Mulao nationality is one of the 55 minorities with population of 207,352 according to the fifth national census statistics of China in 2000. Ninety percent of them live in the Luocheng Mulao Autonomous County, Guangxi Zhuang Autonomous Region, People's Republic of China. The records show that the history of this minority can be traced back to the Jin Dynasty (AD 265-420). It is believed that the Mulao people are the descendants of the ancient Baiyue tribe in south China and ethnically related to the neighboring ethnic groups. A previous study has shown that the genetic relationship between Mulao nationality and other minorities in Guangxi was much closer than that between Mulao and Han or Uighur nationlity [32]. To the best of our knowledge, however, the serum lipid profiles and the association of genetic polymorphisms and serum lipid levels have not been previously reported in this population. Therefore, the aim of the present study was to detect the association of *GALNT2 *rs2144300 and rs4846914 SNPs and several environmental factors with serum lipid profiles in the Mulao and Han populations.

## Materials and Methods

### Study populations

A total of 775 unrelated subjects of Mulao nationality who reside in Luocheng Mulao Autonomous County, Guangxi Zhuang Autonomous Region, People's Republic of China were randomly selected from our stratified randomized cluster samples. The ages of the subjects ranged from 15 to 80 years, with an average age of 52.20 ± 11.68 years. There were 310 males (40.0%) and 465 females (60.0%). All subjects were rural agricultural workers. During the same period, a total of 699 people of Han nationality who reside in the same villages were also randomly selected from our previous stratified randomized cluster samples. The average age of the subjects was 51.42 ± 15.34 years (range 15 to 80). There were 266 men (38.1%) and 433 women (61.9%). All of them were also rural agricultural workers. The subjects had no evidence of diseases related to atherosclerosis, CAD and diabetes. None of them were using lipid-lowering medication such as statins or fibrates when the blood sample was taken. The study design was approved by the Ethics Committee of the First Affiliated Hospital, Guangxi Medical University. Informed consent was obtained from all subjects after they received a full explanation of the study.

### Epidemiological survey

The survey was carried out using internationally standardized methods, following a common protocol [33]. All participants underwent a complete history, physical examination, and laboratory assessment of cardiovascular risk factors, including cigarette smoking, family history of myocardial infarction, blood pressure, presence of diabetes mellitus. Information on demographics, socioeconomic status, and lifestyle factors was collected with standardized questionnaires. The alcohol information included questions about the number of liangs (about 50 g) of rice wine, corn wine, rum, beer, or liquor consumed during the preceding 12 months. Alcohol consumption was categorized into groups of grams of alcohol per day: ≤ 25 and > 25. Smoking status was categorized into groups of cigarettes per day: ≤ 20 and > 20. At the physical examination, several parameters including body height, weight, and waist circumference were measured. Sitting blood pressure was measured three times with the use of a mercury sphygmomanometer after the subjects had a 5-minute rest, and the average of the three measurements was used for the level of blood pressure. Systolic blood pressure was determined by the first Korotkoff sound, and diastolic blood pressure by the fifth Korotkoff sound. Body weight, to the nearest 50 grams, was measured using a portable balance scale. Subjects were weighed without shoes and in a minimum of clothing. Body height was measured, to the nearest 0.5 cm, using a portable steel measuring device. From these two measurements body mass index (BMI, kg/m^2^) was calculated.

### Biochemical analysis

A venous blood sample of 5 mL was obtained from all subjects after at least 12 hours of fasting. A part of the sample (2 mL) was collected into glass tubes and used to determine serum lipid levels. Another part of the sample (3 mL) was transferred to tubes with anticoagulate solution (4.80 g/L citric acid, 14.70 g/L glucose, and 13.20 g/L tri-sodium citrate) and used to extract deoxyribonucleic acid (DNA). Measurements of serum TC, TG, HDL-C, and LDL-C levels in the samples were performed by enzymatic methods with commercially available kits (RANDOX Laboratories Ltd., Ardmore, Diamond Road, Crumlin Co. Antrim, United Kingdom, BT29 4QY; Daiichi Pure Chemicals Co., Ltd., Tokyo, Japan). Serum ApoA1 and ApoB levels were detected by the immunoturbidimetric immunoassay using a commercial kit (RANDOX Laboratories Ltd.). All determinations were performed with an autoanalyzer (Type 7170A; Hitachi Ltd., Tokyo, Japan) in the Clinical Science Experiment Center of the First Affiliated Hospital, Guangxi Medical University [7,8].

### DNA preparation and genotyping

Total genomic DNA was isolated from peripheral blood leukocytes using the phenol-chloroform method [9,10]. The extracted DNA was placed in long-term storage at -80°C. Genotyping of the two SNPs was performed by polymerase chain reaction and restriction fragment length polymorphism (PCR-RFLP). The sequences of the forward and backward primers used for the *GALNT2 *rs2144300 and rs4846914 were 5'-TTGAAGTAGGTGAAGGGGC-3' and 5'-CACATCAACAGCAAAGGGT-3', and 5'-CTGTGCCTTCTGGGACTGCTA-3' and 5'-AGGACTATGAGATGATGGTGG-3' (Sangon, Shanghai, People's Republic of China); respectively. Each reaction system of a total volume of 25 μL, comprised 100 ng (2 μL) of genomic DNA; 1.0 μL of each primer (10 μmo1/L);12.5 μL 2 × Taq PCR MasterMix (constituent: 0.1 U Taq polymerase/μL, 500 μM dNTP each and PCR buffer) and nuclease-free water 8.5 μL. For the amplification, initial denaturation at 95°C for 5 min was followed by 33 cycles of denaturation at 95°C for 45 s, annealing at 59°C for 45 s, and extension at 72°C for 1 min, with final extension at 72°C for 10 min. After electrophoresis on a 2.0% agarose gel with 0.5 μgmL ethidium bromide, the amplifican products were visualized under ultraviolet light. Then each restriction enzyme reaction was performed with 6 μL of amplified DNA; nuclease-free water 7.5 μL and 1 μL of 10 × buffer solution; and restriction ezyme (5 U *MN1I *for rs2144300 and 5 U *HpyF3I *for rs4846914) in a total volume of 15 μL digested at 37°C overnight. After restriction enzyme digestion of the amplified DNA, the digestive products were separated by electrophoresis on sepharose gel. The length of each digested DNA fragment was determined by comparing migration of a sample with that of standard DNA marker. Stained with ethidium bromide, the gel was visualized under ultraviolet light and photographed. Genotypes were scored by an experienced reader blinded to epidemiological data and serum lipid levels.

### DNA sequencing

Twelve samples (each genotype in two) detected by the PCR-RFLP were also confirmed by direct sequencing. The PCR products were purified by low melting point gel electrophoresis and phenol extraction, and then the DNA sequences were analyzed by using an ABI Prism 3100 (Applied Biosyatems) in Shanghai Sangon Biological Engineering Technology & Services Co., Ltd., People's Republic of China.

### Diagnostic criteria

The normal values of serum TC, TG, HDL-C, LDL-C, ApoA1, ApoB levels and the ratio of ApoA1 to ApoB in our Clinical Science Experiment Center were 3.10-5.17, 0.56-1.70, 1.16-1.42, 2.70-3.10 mmol/L, 1.20-1.60, 0.80-1.05 g/L, and 1.00-2.50; respectively. The individuals with TC > 5.17 mmol/L and/or TG > 1.70 mmol/L were defined as hyperlipidemic [7,8]. Hypertension was diagnosed according to the criteria of 1999 World Health Organization-International Society of Hypertension Guidelines for the management of hypertension [34,35]. The diagnostic criteria of overweight and obesity were according to the Cooperative Meta-analysis Group of China Obesity Task Force. Normal weight, overweight and obesity were defined as a BMI < 24, 24-28, and > 28 kg/m^2^; respectively [36].

### Statistical analysis

Epidemiological data were recorded on a pre-designed form and managed with Excel software. The statistical analyses were done with the statistical software package SPSS 13.0 (SPSS Inc., Chicago, Illinois). Quantitative variables were expressed as mean ± standard deviation (serum TG levels were presented as medians and interquartile ranges). Qualitative variables were expressed as percentages. Allele frequency was determined via direct counting, and the standard goodness-of-fit test was used to test the Hardy-Weinberg equilibrium. Difference in genotype distribution between the groups was obtained using the chi-square test. The difference in general characteristics between two ethnic groups was tested by the Student's unpaired *t*-test. The association of genotypes and serum lipid parameters was tested by analysis of covariance (ANCOVA). Age, sex, BMI, blood pressure, alcohol consumption, and cigarette smoking were adjusted for the statistical analysis. In order to assess the association of serum lipid levels with genotypes (rs2144300: CT/TT = 0, CC = 1; rs4846914: GA/AA = 0, GG = 1) and several environment factors, multivariable linear regression analyses with stepwise modeling were also performed in the combined population of Mulao and Han, Mulao, Han, males, and females; respectively. A *P *value of less than 0.05 was considered statistically significant.

## Results

### General and biochemical characteristics

The general and biochemical characteristics between Mulao and Han nationalities are detailed in Table 1. The levels of body height, LDL-C, ApoB, and the percentages of subjects who consumed alcohol were higher but the levels of BMI were lower in Mulao nationality than in Han ethnic group (*P *< 0.05-0.001). There were no significant differences in the levels of age, weight, waist circumference, systolic blood pressure, diastolic blood pressure, pulse pressure, blood glucose, TC, TG, HDL-C, ApoA1; the ratio of ApoA1 to ApoB; the percentages of subjects who smoked cigarettes; and the ratio of male to female between the two ethnic groups (*P *> 0.05 for all).

**Table 1:** Comparison of demographics, lifestyle and serum lipid levels between the Mulao and Han populations

Parameter	Mulao	Han	*t *(χ^2^)	*P*
Number	775	699		
Male/female	310/465	266/433	0.584	0.445
Age (years)	52.20 ± 11.68	51.42 ± 15.34	1.187	0.277
Height (cm)	155.08 ± 7.36	154.11 ± 7.88	5.863	0.016
Weight (kg)	53.03 ± 8.82	53.14 ± 8.80	0.066	0.798
Body mass index (kg/m^2^)	21.99 ± 2.96	22.37 ± 3.40	4.961	0.026
Waist circumference (cm)	75.52 ± 8.42	75.21 ± 7.94	0.529	0.467
Cigarette smoking (n %)				
Nonsmoker	600(77.4)	525(75.1)		
≤ 20 Cigarettes/day	144(18.6)	154(22.0)		
> 20 Cigarettes/day	31(4.0)	20(2.9)	3.800	0.150
Alcohol consumption [n (%)]				
Nondrinker	603(77.8)	565(80.8)		
≤ 25 g/day	56(7.2)	66(9.4)		
> 25 g/day	116(15.0)	68(9.7)	10.688	0.005
Systolic blood pressure (mmHg)	128.54 ± 21.06	129.74 ± 19.03	1.302	0.254
Diastolic blood pressure (mmHg)	81.44 ± 11.98	82.35 ± 10.81	2.338	0.126
Pulse pressure (mmHg)	47.10 ± 15.19	47.38 ± 14.43	0.136	0.713
Blood glucose (mmol/L)	6.07 ± 1.69	6.03 ± 1.61	0.231	0.631
Total cholesterol (mmol/L)	5.06 ± 1.31	4.99 ± 1.11	0.191	0.275
Triglyceride (mmol/L)	1.10 (0.80)	1.05 (0.91)	-0.502	0.616
HDL-C (mmol/L)	1.75 ± 0.42	1.73 ± 0.54	0.412	0.521
LDL-C (mmol/L)	2.98 ± 0.95	2.87 ± 0.89	4.786	0.029
Apolipoprotein(Apo)A1 (g/L)	1.35 ± 0.39	1.34 ± 0.26	0.332	0.565
ApoB (g/L)	0.98 ± 0.55	0.85 ± 0.21	37.579	0.000
ApoA1/ApoB	1.61 ± 0.77	1.66 ± 0.49	1.796	0.160

HDL-C, high-density lipoprotein cholesterol; LDL-C, low-density lipoprotein cholesterol. The values of triglyceride were presented as median (interquartile range). The difference between the two ethnic groups was determined by the Wilcoxon-Mann-Whitney test.

### Electrophoresis and genotyping

**Figure 1:**
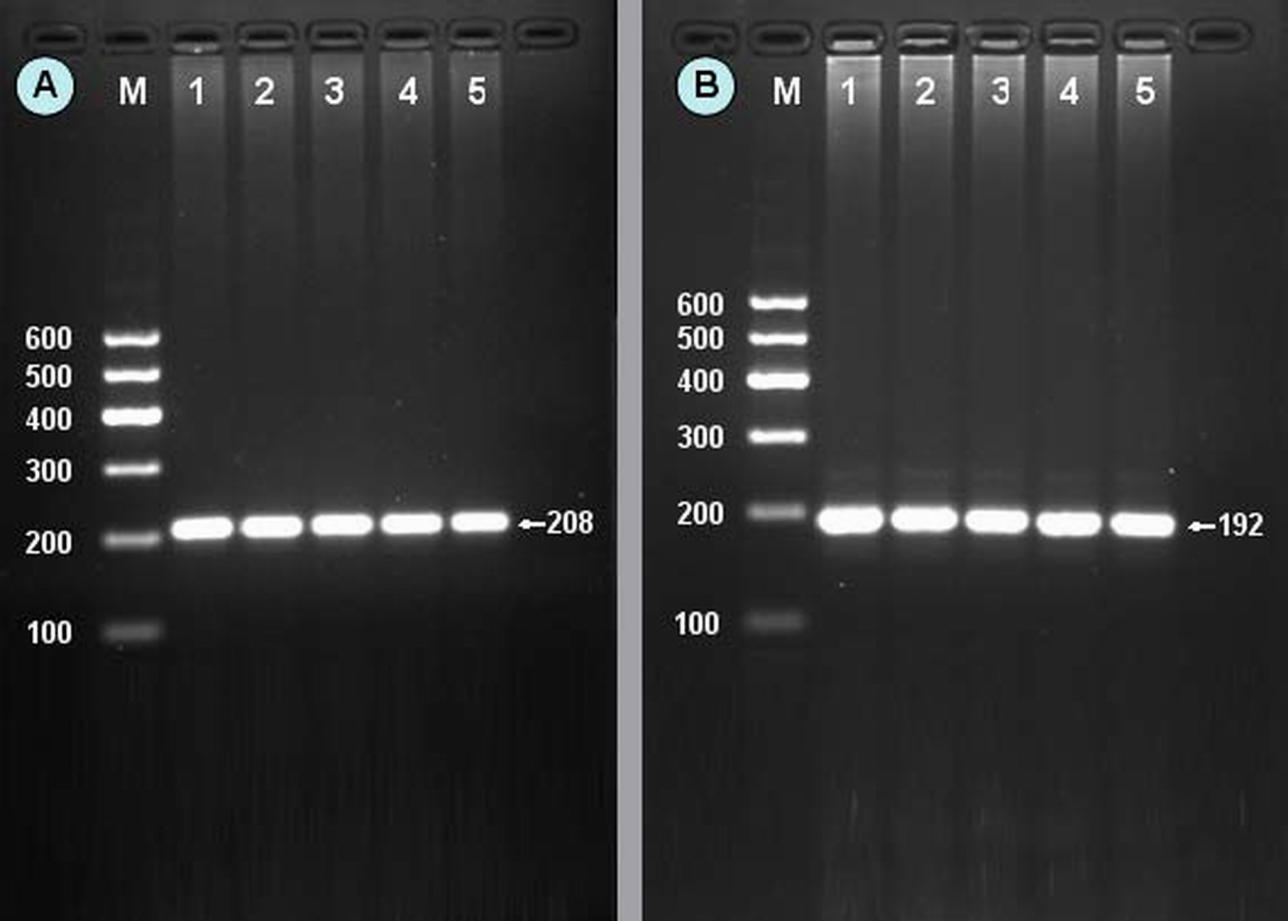
**Electrophoresis of PCR products of the samples**. (A) *GALNT2 *rs2144300. Lane M, 100 bp marker ladder; lanes 1-5, samples. The 208 bp bands are the PCR products. (B) *GALNT2 *rs4846914. Lane M, 100 bp marker ladder; lanes 1-5, samples. The 192 bp bands are the PCR products.

**Figure 2:**
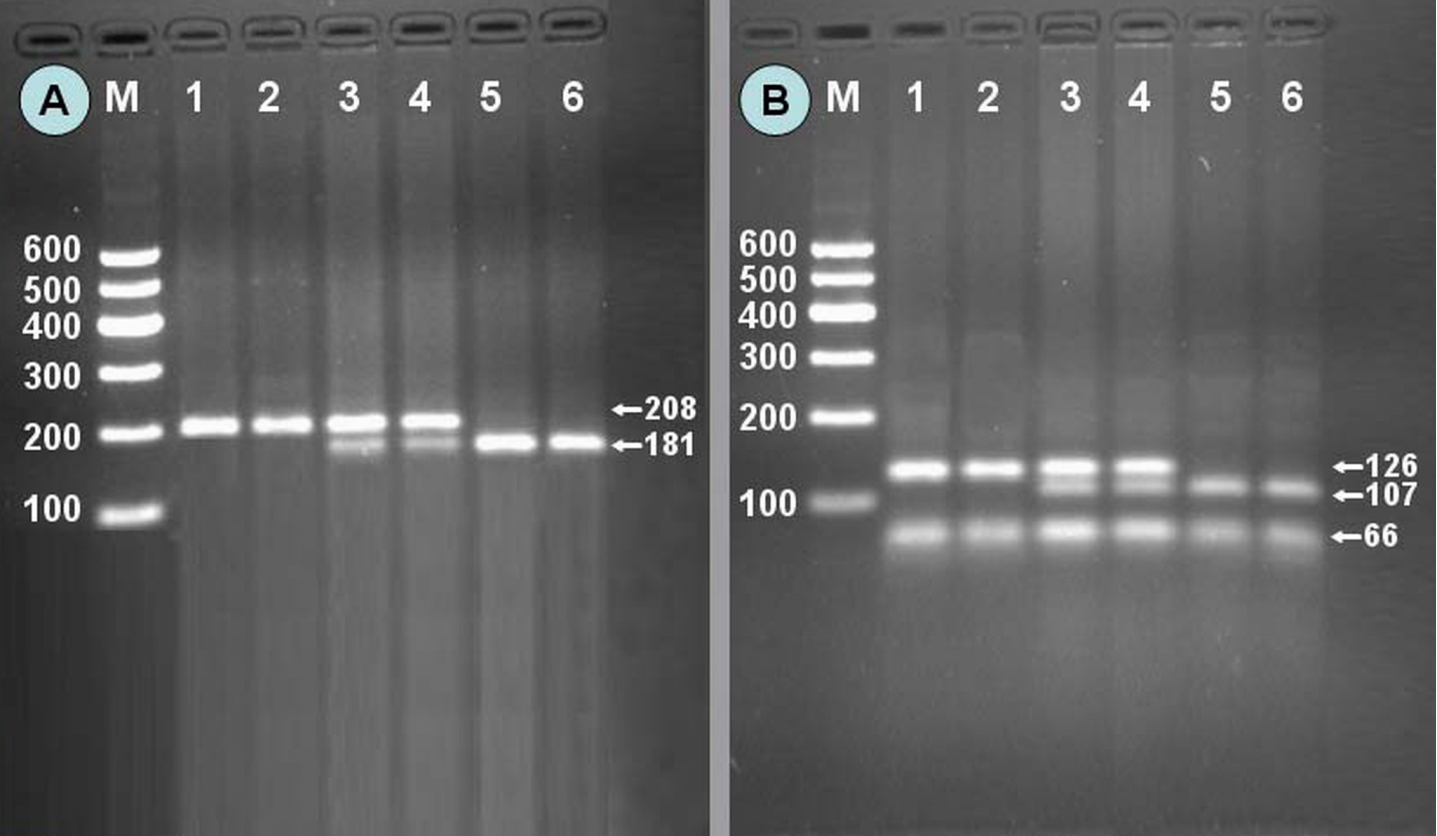
**Genotyping of the *GALNT2 *rs2144300 and rs4846914 polymorphisms**. (A) *GALNT2 *rs2144300. Lane M, 100 bp marker ladder; lanes 1 and 2, CC genotype (208 bp); lanes 3 and 4, CT genotype (208-, 181- and 27-bp); and lanes 5 and 6, TT genotype (181- and 27-bp). (B) *GALNT2 *rs4846914. Lane M, 100 bp marker ladder; lanes 1 and 2, AA genotype (126- and 66-bp); lanes 3 and 4, GA genotype (126-, 107-, 66- and 19-bp); and lanes 5 and 6, GG genotype (107-, 66- and 19-bp). The 27- and 19-bp fragments were invisible in the gel owing to their fast migration speed.

After the genomic DNA of the samples was amplified by PCR and imaged by agarose gel electrophoresis, the PCR products of 208 bp (rs2144300) and 192 bp (rs4846914) nucleotide sequences could be seen in the samples (Figure 1). The CC (208 bp), CT (208-, 181- and 27-bp) and TT (181- and 27-bp) genotypes of rs2144300 are shown in Figure 2A. The AA (126- and 66-bp), GA (126-, 107-, 66-, and 19-bp), and GG (107-, 66-, and 19-bp) genotypes of rs4846914 are shown in Figure 2B. The 27- and 19-bp fragments were invisible in the gel owing to their fast migration speed.

### Results of sequencing

**Figure 3:**
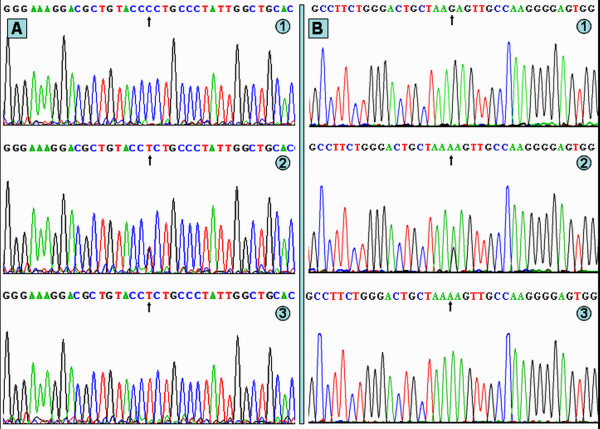
**A part of the nucleotide sequences of *GALNT2 *rs2144300 and rs4846914 SNPs**. (A) *GALNT2 *rs2144300 SNP: (1) CC genotype, (2) CT genotype, (3) TT genotype. (B) *GALNT2 *rs4846914 SNP: (1) GG genotype, (2) GA genotype, (3) AA genotype.

The results were shown as CC, CT and TT genotypes of rs2144300 SNP and GG, GA and AA genotypes of rs4846914 SNP by PCR-RFLP, the genotypes were also confirmed by sequencing (Figure 3); respectively.

### Genotypic and allelic frequencies

The genotypic frequencies of both SNPs were all in Hardy-Weinberg equilibrium. The C and T allele frequencies of *GALNT2 *rs2144300 were 79.10% and 20.90% in Mulao, and 80.54% and 19.46% in Han (*P *> 0.05); respectively. The frequencies of CC, CT and TT genotypes were 63.35%, 31.48% and 5.16% in Mulao, and 63.95%, 33.19% and 2.86% in Han (*P *> 0.05); respectively. The G and A allele frequencies of *GALNT2 *rs4846914 were 78.38% and 21.62% in Mulao, and 75.50% and 24.50% in Han (*P *> 0.05); respectively. The frequencies of GG, GA and AA genotypes were 61.47%, 33.82% and 4.71% in Mulao, and 56.24%, 38.52% and 5.24% in Han (*P *> 0.05; Table 2); respectively.

**Table 2:** Comparison of the genotypic and allelic frequencies of *GALNT2 *rs2144300 and rs4846914 between the Mulao and Han populations

			Genotype [n (%)]			Allele [n (%)]	
SNP	Group	n					
			AA	AB	BB	A	B
***GALNT2 *rs2144300**	Mulao	775	491(63.35)	244(31.48)	40(5.16)	1226(79.10)	324(20.90)
	Han	699	447(63.95)	232(33.19)	20(2.86)	1126(80.54)	272(19.46)
	χ^2^	-	5.075			0.954	
	*P*	-	0.079			0.329	
	Mulao/male	310	208(67.10)	85(27.42)	17(5.48)	501(80.81)	119(19.19)
	Mulao/female	465	283(60.86)	159(34.19)	23(4.95)	725(77.96)	205(22.04)
	χ^2^	-	3.957			1.827	
	*P*	-	0.138			0.177	
	Han/male	266	158(59.40)	100(37.59)	8(3.01)	416(78.20)	116(21.80)
	Han/female	433	289(66.75)	132(30.48)	12(2.77)	710(81.99)	156(18.01)
	χ^2^	-	3.931			3.022	
	*P*	-	0.140			0.082	
***GALNT2 *rs4846914**	Mulao	680	418(61.47)	230(33.82)	32(4.71)	1066(78.38)	294(21.62)
	Han	649	365(56.24)	250(38.52)	34(5.24)	980(75.50)	318(24.50)
	χ^2^	-	3.760			3.112	
	*P*	-	0.153			0.078	
	Mulao/male	249	151(60.64)	88(35.34)	10(4.02)	390(78.31)	108(21.69)
	Mulao/female	431	267(61.95)	142(32.95)	22(5.10)	676(78.42)	186(21.58)
	χ^2^	-	0.709			0.002	
	*P*	-	0.702			0.962	
	Han/male	260	159(61.15)	87(33.46)	14(5.39)	405(77.88)	115(22.12)
	Han/female	389	206(53.96)	163(41.90)	20(5.14)	575(73.91)	203(26.09)
	χ^2^	-	4.762			2.665	
	*P*	-	0.092			0.103	

Allele A, rs2144300 C and rs4846914 G; Allele B, rs2144300 T and rs4846914 A; Genotype AA, rs2144300 CC and rs4846914 GG; Genotype AB, rs2144300 CT and rs4846914 GA; Genotype BB, rs2144300 TT and rs4846914 AA

### Genotypes and serum lipid levels

As shown in Table 3, the levels of TG in Mulao were different among the CC, CT and TT genotypes of rs2144300 (*P *< 0.05). The subjects with TT genotype had lower serum TG levels than the subjects with CC genotype, these results were found in females (*P *< 0.01) but not in males. The levels of TG, HDL-C, ApoA1, ApoB, and the ratio of ApoA1 to ApoB in Han were different between the CC and CT/TT genotypes of rs2144300 in males (*P *< 0.05-0.001), the subjects with CT/TT genotype had lower serum TG and ApoB levels and higher serum HDL-C, ApoA1 levels, and the ratio of ApoA1 to ApoB than the subjects with CC genotype. The levels of LDL-C and ApoB in Han were different between the CC and CT/TT genotypes of rs2144300 in females (*P *< 0.01 and *P *< 0.05; respectively), the subjects with CT/TT genotype had higher serum LDL-C and ApoB levels than the subjects with CC genotype.

**Table 3:** The *GALNT2 *rs2144300 and rs4846914 genotypes and serum lipid levels between the Mulao and Han populations

SNP	Genotype	n	TC (mmol/L)	TG (mmol/L)	HDL-C (mmol/L)	LDL-C (mmol/L)	ApoA1 (g/L)	ApoB (g/L)	ApoA1/ ApoB
*GALNT2 *rs2144300									
Mulao	CC	491	5.06 ± 1.39	1.15(0.84)	1.74 ± 0.42	2.97 ± 0.96	1.35 ± 0.39	0.99 ± 0.55	1.58 ± 0.77
	CT	244	5.05 ± 1.16	1.05(0.83)	1.77 ± 0.43	2.97 ± 0.92	1.35 ± 0.40	0.96 ± 0.54	1.67 ± 0.81
	TT	40	5.20 ± 1.01	0.92(0.92)	1.73 ± 0.38	3.11 ± 0.96	1.32 ± 0.40	0.97 ± 0.47	1.51 ± 0.58
	*F*	-	0.542	6.874	0.505	1.037	0.115	0.024	0.947
	*P*	-	0.582	0.032	0.603	0.355	0.892	0.976	0.388
Mulao/male	CC	208	5.12 ± 1.42	1.17(1.11)	1.75 ± 0.42	2.86 ± 0.92	1.39 ± 0.41	1.05 ± 0.64	1.56 ± 0.67
	CT/TT	102	5.39 ± 0.97	1.12(1.20)	1.73 ± 0.42	3.06 ± 0.86	1.37 ± 0.35	1.10 ± 0.62	1.46 ± 0.60
	*F*	-	3.107	-0.118	0.000	1.859	0.003	0.721	0.931
	*P*	-	0.079	0.906	0.986	0.174	0.959	0.397	0.335
Mulao/female	CC	283	5.02 ± 1.38	1.14 (0.65)	1.72 ± 0.42	3.06 ± 0.98	1.32 ± 0.37	0.95 ± 0.48	1.61 ± 0.84
	CT/TT	182	4.89 ± 1.20	0.99(0.60)	1.79 ± 0.44	2.95 ± 0.96	1.33 ± 0.42	0.88 ± 0.47	1.75 ± 0.84
	*F*	-	0.354	-2.851	0.540	0.127	0.091	0.876	1.224
	*P*	-	0. 552	0.004	0.463	0.722	0.763	0.350	0.269
Han	CC	447	4.97 ± 1.02	1.00 (0.90)	0.73 ± 0.60	2.83 ± 0.86	1.32 ± 0.24	0.84 ± 0.20	1.64 ± 0.46
	CT	232	5.05 ± 1.27	1.07(0.91)	1.73 ± 0.44	2.90 ± 0.96	1.36 ± 0.28	0.85 ± 0.22	1.68 ± 0.51
	TT	20	4.89 ± 1.51	1.10(1.12)	1.79 ± 0.42	3.28 ± 0.67	1.41 ± 0.36	0.93 ± 0.15	1.57 ± 0.52
	*F*	-	1.452	0.394	0.403	0.309	2.191	1.715	3.572
	*P*	-	0.235	0.821	0.668	0.734	0.113	0.181	0.029
Han/male	CC	158	5.25 ± 0.96	1.32(1.17)	1.61 ± 0.42	3.01 ± 0.81	1.31 ± 0.26	0.93 ± 0.20	1.47 ± 0.41
	CT/TT	108	5.21 ± 1.32	1.09 (0.93)	1.71 ± 0.42	2.87 ± 0.92	1.40 ± 0.30	0.89 ± 0.20	1.64 ± 0.47
	*F*	-	0.120	-2.085	4.795	1.156	6.432	4.478	13.073
	*P*	-	0.729	0.040	0.029	0.283	0.012	0.035	0.000
Han/female	CC	289	4.81 ± 1.02	0.91(0.78)	1.79 ± 0.66	2.75 ± 0.87	1.32 ± 0.24	0.80 ± 0.19	1.73 ± 0.46
	CT/TT	144	4.89 ± 1.25	1.06(0.95)	1.75 ± 0.46	2.96 ± 0.97	1.33 ± 0.27	0.84 ± 0.23	1.70 ± 0.54
	*F*	-	1.879	-1.683	1.212	7.387	0.043	5.929	1.424
	*P*	-	0.171	0.092	0.272	0.007	0.836	0.018	0.233
*GALNT2 *rs4846914									
Mulao	GG	365	5.03 ± 1.18	1.12(0.83)	1.75 ± 0.43	2.99 ± 0.89	1.36 ± 0.38	0.99 ± 0.54	1.58 ± 0.61
	GA	250	5.02 ± 1.11	1.07(0.84)	1.76 ± 0.44	2.96 ± 0.86	1.32 ± 0.43	0.95 ± 0.52	1.64 ± 0.77
	AA	34	5.40 ± 0.84	1.05(0.79)	1.72 ± 0.37	3.27 ± 0.88	1.32 ± 0.38	1.05 ± 0.49	1.42 ± 0.59
	*F*	-	1.810	1.253	0.089	1.911	0.787	0.343	1.430
	*P*	-	0.164	0.535	0.915	0.149	0.456	0.710	0.240
Mulao/male	GG	159	4.97 ± 1.02	1.16(1.18)	1.75 ± 0.46	2.86 ± 0.79	1.38 ± 0.43	1.00 ± 0.59	1.58 ± 0.65
	GA/AA	101	5.23 ± 0.97	1.21(1.04)	1.74 ± 0.41	3.08 ± 0.78	1.37 ± 0.39	1.05 ± 0.57	1.53 ± 0.70
	*F*	-	5.626	-0.553	0.012	4.170	0.007	0.305	0.108
	*P*	-	0.018	0.580	0.913	0.042	0.935	0.582	0.743
Mulao/female	GG	206	5.08 ± 1.29	1.12(0.62)	1.75 ± 0.40	3.09 ± 0.95	1.35 ± 0.34	0.98 ± 0.50	1.57 ± 0.57
	GA/AA	183	4.93 ± 1.14	1.02(0.71)	1.77 ± 0.44	2.96 ± 0.91	1.29 ± 0.44	0.91 ± 0.48	1.66 ± 0.80
	*F*	-	1.434	-1.597	0.117	1.419	2.219	0.839	0.530
	*P*	-	0. 232	0.110	0.773	0.234	0.137	0.360	0.467
Han	GG	418	4.97 ± 1.01	1.00(0.80)	1.70 ± 0.40	2.85 ± 0.86	1.31 ± 0.24	0.84 ± 0.19	1.63 ± 0.45
	GA	230	5.01 ± 1.15	1.06(0.95)	1.80 ± 0.72	2.84 ± 0.86	1.37 ± 0.26	0.84 ± 0.20	1.71 ± 0.50
	AA	32	5.16 ± 1.34	1.40(1.22)	1.62 ± 0.40	3.41 ± 0.72	1.32 ± 0.31	0.96 ± 0.17	1.43 ± 0.47
	*F*	-	0.261	2.716	3.415	4.060	2.658	3.255	3.901
	*P*	-	0.770	0.257	0.033	0.018	0.071	0.039	0.021
Han/male	GG	151	5.23 ± 0.95	1.16(1.05)	1.63 ± 0.38	3.00 ± 0.81	1.32 ± 0.23	0.92 ± 0.20	1.50 ± 0.41
	GA/AA	98	5.19 ± 1.38	1.15(1.23)	1.67 ± 0.45	2.85 ± 0.93	1.40 ± 0.33	0.89 ± 0.21	1.63 ± 0.51
	*F*	-	0.136	-0.149	0.253	0.532	2.429	0.762	3.096
	*P*	-	0.713	0.881	0.615	0.467	0.120	0.384	0.080
Han/female	GG	267	4.83 ± 1.02	0.96(0.73)	1.74 ± 0.41	2.77 ± 0.88	1.31 ± 0.24	0.80 ± 0.18	1.71 ± 0.45
	GA/AA	164	4.94 ± 1.03	1.04(0.95)	1.84 ± 0.79	2.94 ± 0.82	1.34 ± 0.21	0.84 ± 0.19	1.70 ± 0.50
	*F*	-	0.247	-1.122	2.904	1.735	1.389	0.684	0.575
	*P*	-	0.619	0.262	0.089	0.188	0.239	0.409	0.449

SNP, single nucleotide polymorphism; TC, total cholesterol; TG, triglyceride; HDL-C, high-density lipoprotein cholesterol; LDL-C, low-density lipoprotein cholesterol; ApoA1, apolipoprotein A1; ApoB, apolipoprotein B; ApoA1/ApoB, the ratio of apolipoprotein A1 to apolipoprotein B;

The values of triglyceride were presented as median (interquartile range). The difference among the genotypes was determined by the Kruskal-Wallis test or the Wilcoxon-Mann-Whitney test.

The levels of TC and LDL-C in Mulao were different between the GG and GA/AA genotypes of rs4846914 in males (*P *< 0.05 for each) but not in females, the subjects with GA/AA genotype had higher serum TC and LDL-C levels. The levels of HDL-C, LDL-C, ApoB, and the ratio of ApoA1 to ApoB in Han were different among the GG, GA, and AA genotypes of rs4846914 (*P *< 0.05 for all), the subjects with AA genotype had higher serum LDL-C and ApoB levels and lower serum HDL-C levels and the ratio of ApoA1 to ApoB than the subjects with GG genotype.

### Risk factors for serum lipid parameters

The correlation between the genotypes of *GALNT2 *rs2144300 and rs4846914 and serum lipid parameters in Mulao and Han is shown in Table 4. The levels of TC in Mulao were correlated with the genotypes of rs4846914 in males (*P *< 0.05) but not in females. The levels of ApoA1 in Han were correlated with the genotypes of both rs2144300 and rs4846914 SNPs, and the levels of HDL-C, ApoB, and the ratio of ApoA1 to ApoB were associated with the genotypes of rs2144300 in males (*P *< 0.05-0.001). The levels of LDL-C in Han were correlated with the genotypes of rs4846914 in females (*P *< 0.05).

**Table 4:** Correlation between the *GALNT2 *rs2144300 and rs4846914 genotypes and serum lipid levels in the Mulao and Han populations

Lipid	Genotype	Unstandardized coefficient	Std. error	Standardized coefficient	*t*	*P*
Mulao and Han						
LDL-C	rs4846914 genotype	-0.354	0.133	-0.199	-2.668	0.008
Han						
HDL-C	rs4846914 genotype	0.079	0.041	0.072	1.986	0.048
ApoA1	rs4846914 genotype	0.039	0.019	0.076	2.068	0.039
Mulao/male						
TC	rs4846914 genotype	0.292	0.127	0.141	2.302	0.022
Han/male						
HDL-C	rs2144300 genotype	-0.397	0.141	-0.527	-2.816	0.005
ApoA1	rs2144300 genotype	-0.085	0.027	-0.172	-3.132	0.002
	rs4846914 genotype	0.055	0.028	0.113	1.978	0.049
ApoB	rs2144300 genotype	0.043	0.021	0.108	2.057	0.041
ApoA1/ApoB	rs2144300 genotype	-0.165	0.043	-0.204	-3.816	0.000
Han/female						
LDL-C	rs4846914 genotype	0.162	0.065	0.111	2.493	0.013

TC, total cholesterol; HDL-C, high-density lipoprotein cholesterol; LDL-C, low-density lipoprotein cholesterol; ApoA1, apolipoprotein A1; ApoB, apolipoprotein B; ApoA1/ApoB, the ratio of apolipoprotein A1 to apolipoprotein B

Serum lipid parameters were also correlated with several environment factors such as age, gender, alcohol consumption, cigarette smoking, blood pressure, blood glucose, BMI, and waist circumference in both ethnic groups (Table 5).

**Table 5:** The environmental risk factors for serum lipid parameters in the Mulao and Han populations

Lipid	Risk factor	Unstandardized coefficient	Std. error	Standardized coefficient	*t*	*P*
Mulao and Han						
TC	Age	0.015	0.002	0.167	6.397	0.000
	Waist circumference	0.012	0.005	0.080	2.178	0.030
	Alcohol consumption	0.125	0.053	0.070	2.380	0.017
	Diastolic blood pressure	0.008	0.003	0.073	2.704	0.007
	Cigarette smoking	0.153	0.069	0.065	2.227	0.026
	Body mass index	0.029	0.014	0.075	2.075	0.038
TG	Waist circumference	0.066	0.007	0.251	9.853	0.000
	Alcohol consumption	0.347	0.091	0.110	3.823	0.000
	Blood glucose	0.103	0.033	0.079	3.146	0.002
	Cigarette smoking	0.320	0.119	0.077	2.690	0.007
HDL-C	Waist circumference	-0.01	0.002	-0.167	-4.49	0.000
	Alcohol consumption	0.120	0.022	0.170	5.464	0.000
	Age	0.003	0.001	0.097	3.786	0.000
	Gender	0.108	0.031	0.109	3.440	0.001
	Body mass index	-0.012	0.006	-0.079	-2.165	0.031
LDL-C	Age	0.012	0.002	0.182	6.923	0.000
	Body mass index	0.046	0.008	0.160	6.068	0.000
	Alcohol consumption	-0.104	0.035	-0.077	-2.970	0.003
	Diastolic blood pressure	0.005	0.002	0.058	2.118	0.034
ApoA1	Alcohol consumption	0.106	0.013	0.216	8.242	0.000
	Waist circumference	-0.004	0.001	-0.107	-4.079	0.000
	Age	0.002	0.001	0.070	2.696	0.007
ApoB	Waist circumference	0.010	0.001	0.188	7.062	0.000
	Blood glucose	0.024	0.007	0.093	3.592	0.000
	Gender	-0.078	0.023	-0.088	-3.346	0.001
ApoA1/ApoB	Waist circumference	-0.013	0.003	-0.163	-4.402	0.000
	Blood glucose	-0.024	0.010	-0.059	-2.272	0.023
	Body mass index	-0.024	0.007	-0.118	-3.284	0.001
	Gender	0.160	0.042	0.119	3.782	0.000
	Alcohol consumption	0.108	0.029	0.114	3.674	0.000
	Age	-0.002	0.001	-0.051	-1.964	0.050
Han						
TC	Diastolic blood pressure	0.018	0.004	0.171	4.492	0.000
	Waist circumference	0.024	0.005	0.168	4.611	0.000
	Age	0.009	0.003	0.129	3.363	0.001
	Alcohol consumption	0.235	0.063	0.134	3.725	0.000
	Blood glucose	0.071	0.026	0.102	2.785	0.006
TG	Waist circumference	0.073	0.010	0.259	7.165	0.000
	Cigarette smoking	0.747	0.156	0.170	4.799	0.000
	Diastolic blood pressure	0.029	0.008	0.139	3.660	0.000
	Blood glucose	0.179	0.051	0.127	3.474	0.001
	Age	-0.015	0.006	-0.104	-2.717	0.007
HDL-C	Waist circumference	-0.013	0.003	-0.188	-5.038	0.000
LDL-C	Age	0.012	0.002	0.212	5.709	0.000
	Body mass index	0.032	0.013	0.122	2.533	0.012
	Blood glucose	0.043	0.021	0.078	2.071	0.039
	Waist circumference	0.011	0.005	0.098	2.000	0.046
	Cigarette smoking	-0.243	0.081	-0.139	-3.010	0.003
	Gender	-0187	0.086	-0103	-2.174	0.030
ApoA1	Alcohol consumption	0.109	0.018	0.268	6.609	0.000
	Body mass index	-0.010	0.003	-0.135	-3.625	0.000
	Gender	0.093	0.026	0.174	3.506	0.000
	Cigarette smoking	0.083	0.024	0.163	3.415	0.001
ApoB	Waist circumference	0.006	0.001	0.228	5.110	0.000
	Systolic blood pressure	0.003	0.000	0.095	2.122	0.034
	Blood glucose	0.025	0.004	0.190	5.640	0.000
	Alcohol consumption	0.048	0.011	0.149	4.390	0.000
	Body mass index	0.008	0.003	0.134	2.966	0.003
	Pulse pressure	-.002	0.001	-0.128	-2.162	0.031
ApoA1/ApoB	Waist circumference	-0.010	0.003	-0.166	-3.507	0.000
	Body mass index	-0.027	0.007	-0.185	-3.961	0.000
	Age	-0.003	0.001	-0.096	-2.681	0.000
	Gender	0.224	0.046	0.222	4.869	0.000
	Cigarette smoking	0.186	0.043	0.193	4.309	0.000
	Blood glucose	-0.023	0.11	-0.075	-2.080	0.038
Mulao						
TC	Age	0.017	0.004	0.155	4.412	0.000
	Body mass index	0.056	0.016	0.126	3.587	0.000
	Cigarette smoking	0.250	0.088	0.100	2.845	0.005
TG	Waist circumference	0.058	0.008	0.239	6.886	0.000
	Alcohol consumption	0.479	0.097	0.171	4.920	0.000
HDL-C	Body mass index	-0.024	0.008	-0.166	-3.068	0.002
	Alcohol consumption	0.091	0.020	0.158	4.541	0.000
	Age	0.004	0.001	0.111	3.239	0.001
	Waist circumference	-0.007	0.003	-0.144	-2.630	0.009
LDL-C	Age	0.011	0.003	0.133	3.715	0.000
	Body mass index	0.039	0.012	0.120	3.317	0.001
	Alcohol consumption	-0.152	0.046	-0.117	-3.270	0.001
	Diastolic blood pressure	0.006	0.003	0.075	2.002	0.046
ApoA1	Alcohol consumption	0.105	0.019	0.196	5.449	0.000
	Waist circumference	-0.004	0.002	-0.091	-2.541	0.011
ApoB	Waist circumference	0.010	0.002	0.149	4.049	0.000
	Gender	-0.090	0.041	-0.080	-2.200	0.028
	Blood glucose	0.025	0.012	0.077	2.145	0.032
ApoA1/ApoB	Waist circumference	-0.020	0.003	-0.223	-6.321	0.000

TC, total cholesterol; TG, triglyceride; HDL-C, high-density lipoprotein cholesterol; LDL-C, low-density lipoprotein cholesterol; ApoA1, apolipoprotein A1; ApoB, apolipoprotein B; ApoA1/ApoB, the ratio of apolipoprotein A1 to apolipoprotein B

## Discussion

The results of the present study show that the levels of serum LDL-C and ApoB were higher in Mulao than in Han nationalities. There were no significant differences in the levels of serum TC, TG, HDL-C, ApoA1, and the ratio of ApoA1 to ApoB between the two ethnic groups. It is well known that dyslipidemia is a multifactorial origin, including environmental factors such as demographics, diet, alcohol consumption, cigarette smoking, obesity, exercise, hypertension [7,8]; genetic factors such as variants in genes coding for proteins; and their interactions [9,10]. For Mulao people, engagements were family-arranged in childhood, usually with the girl being four or five years older than the boy. There was a preference for marriage to mother's brother's daughter. Engagement and marriage were marked by bride-wealth payments. Marriage ceremonies were held when the girl reached puberty. She remained with her natal family until her first child was born. Till then she was free to join the young men and women who came together for responsive singing, flirtations, and courtships at festival times. Divorce and remarriage were permitted, with little restriction. The two-generation household is the most common unit of residence. Households are under the control of the father, and divide when the sons marry, with only the youngest son remaining with the parents. Daughters could not inherit property, and if there were no sons the property went to a nephew or lineage cousin's son. Therefore, we guessed that the hereditary characteristics and some lipid metabolism-related gene polymorphisms in this population may be different from those in Han nationality.

The genotypic and allelic frequencies of *GALNT2 *rs2144300 and rs4846914 SNPs in diverse racial/ethnic groups are not well known. In the present study, we showed that there were no significant differences in the genotypic and allelic frequencies of the two SNPs between the Mulao and Han populations, or between the males and females in both ethnic groups. These findings are similar to the results of a previous study in patients with stroke and control group [29]. Polgár et al. [29] showed that the allelic frequency of *GALNT2 *rs4846914 in patients with stroke did not significantly differ from that in control group. Also, the genotypic frequencies were similar to frequencies obtained in other populations [15,17] and to data available in the International HapMap Project's data-base (http://www.hapmap.org) for the Caucasian CEPH population of European origin [29]. Since there were no observable differences in the genotypic and allelic frequencies of *GALNT2 *rs2144300 and rs4846914 SNPs between the Mulao and Han populations, biologically, Mulao and Han nationalities may be homologous. Also, there were no significant differences in the genotypic and allelic frequencies of *GALNT2 *rs2144300 and rs4846914 SNPs in different races [24].

The potential relationship between the *GALNT2 *polymorphisms and plasma or serum lipid levels in humans has been evaluated in several previous GWA studies. However, previous findings on the association of these SNPs with the changes in plasma lipid levels are inconsistent. Several studies reported that the minor allele of *GALNT2 *polymorphisms was associated with low HDL-C [17,19-25] and high TG blood levels [15,17,24-28]. In the present study, we showed that the subjects with TT genotype of rs2144300 in Mulao nationality had lower serum TG levels than the subjects with CC genotype in females. The participants with CT/TT genotype of rs2144300 in Han had lower TG and ApoB levels and higher HDL-C, ApoA1 levels, and the ratio of ApoA1 to ApoB in males, and higher LDL-C and ApoB levels in females than the participants with CC genotype. The individuals with GA/AA genotype of rs4846914 in Mulao nationality had higher TC and LDL-C levels than the individuals with GG genotype in males. The subjects with AA genotype of rs4846914 in Han had higher LDL-C and ApoB levels and lower HDL-C levels and the ratio of ApoA1 to ApoB than the subjects with GG genotype. The levels of TC in Mulao nationality were correlated with the genotypes of rs4846914 in males. The levels of ApoA1 in Han were correlated with the genotypes of both SNPs, and the levels of HDL-C, ApoB, and the ratio of ApoA1 to ApoB were associated with the genotypes of rs2144300 in males. The levels of LDL-C in Han were correlated with the genotypes of rs4846914 in females. Several GWA and candidate gene studies, however, failed to find a significant association between the *GALNT2 *polymorphisms and plasma lipid levels [29-31]. In a previous study, Polgár et al. [29] could not detect any effect of the *GALNT2 *rs4846914 variant on serum TC and TG levels. The mean blood lipid concentrations did not significantly differ in heterozygous and homozygous carriers from those of the non-carriers in either the stratified stroke subgroups or the overall stroke disease group. In Whitehall II, there was a significant association of the *GALNT2 *polymorphisms and plasma levels of the lipoprotein (a). However, a meta-analysis of the six studies did not confirm any of these findings [31]. This may be because of that the effects of these variants were modest on lipid concentrations or lower statistical power for detecting the association was present [29,37]. Also, different genetic and environmental factors might lead to variable levels of associations in different populations.

It is well known that environmental factors such as dietary patterns, lifestyle, obesity, physical activity, and hypertension are all strongly related with serum lipid levels [7,8]. In the present study, we also showed that serum lipid parameters were correlated with age, sex, alcohol consumption, cigarette smoking, BMI, and blood pressure in both ethnic groups. These data suggest that the environmental factors also play an important role in determining serum lipid levels in our populations. Although rice and corn are the staple foods in both ethnic groups, the people of Mulao nationality like to eat cold foods along with acidic and spicy dishes, so bean soy sauce and pickled vegetables are among their most popular dishes. They also like to eat animal offals which contain abundant saturated fatty acid. For nearly 50 years it has been widely accepted that high-fat diets, particularly those that contain large quantities of saturated fatty acids, raise blood cholesterol concentrations and predispose individuals to cardiovascular disease [38]. We also found that the percentages of subjects who consumed alcohol were higher in Mulao than in Han nationalities. Although the effects of alcohol intake on LDL-C appear to vary by specific patient types or patterns of alcohol intake, and perhaps by population and sex, this topic has been the focus of much recent research [39]. A recent study in older Italian subjects (65-84 years old) has found that alcohol intake increases serum LDL-C levels [40]. Another recent study of Turks also found increases in LDL-C, as well as in ApoB and TG, with alcohol in men, while women had decreased TG and no change in LDL-C or ApoB with alcohol [41].

## Conclusion

The present study shows that the genotypic and allelic frequencies of *GALNT2 *rs2144300 and rs4846914 SNPs were not different between the Mulao and Han populations, or between the males and females in both ethnic groups. But the association of *GALNT2 *polymorphisms and serum lipid levels is different between the two ethnic groups. These differences in the association of *GALNT2 *polymorphisms and serum lipid profiles between the two ethnic groups might partly result from different *GALNT2*-enviromental interactions.

## Competing interests

The authors declare that they have no competing interests.

## Authors' contributions

QL participated in the design, undertook genotyping, and helped to draft the manuscript. RXY conceived the study, participated in the design, carried out the epidemiological survey, collected the samples, and drafted the manuscript. TTY, LM, XLC, XJH, LHHA and DFW carried out the epidemiological survey and collaborated to the genotyping. JZW and WXL carried out the epidemiological survey and collected the samples. All authors read and approved the final manuscript.
